# What, how, and how much do herbivores eat? The Continuous Bite Monitoring method for assessing forage intake of grazing animals

**DOI:** 10.1002/ece3.7477

**Published:** 2021-06-25

**Authors:** Anderson Michel Soares Bolzan, Leonardo S. Szymczak, Laura Nadin, Olivier Jean F. Bonnet, Marcelo O. Wallau, Anibal de Moraes, Renata F. Moraes, Alda L. G. Monteiro, Paulo C. F. Carvalho

**Affiliations:** ^1^ Department of Forage Plants and Agrometeorology Federal University of Rio Grande do Sul Porto Alegre RS Brazil; ^2^ Department of Crop Production and Protection Federal University of Paraná Curitiba PR Brazil; ^3^ Faculty of Veterinary Sciences National University of the Centre of the Buenos Aires Province Tandil Argentina; ^4^ Centre d'Études et de Réalisations Pastorales Alpes‐Méditerranée Digne les Bains France; ^5^ Agronomy Department University of Florida Gainesville FL USA; ^6^ Departament of Animal Science Federal University of Paraná Curitiba PR Brazil

**Keywords:** foraging, grasslands, grazing ecology, herbage intake, Italian ryegrass, short‐term intake rate, tall fescue

## Abstract

Determining herbage intake is pivotal for studies on grazing ecology. Direct observation of animals allows describing the interactions of animals with the pastoral environment along the complex grazing process. The objectives of the study were to evaluate the reliability of the continuous bite monitoring (CBM) method in determining herbage intake in grazing sheep compared to the standard double‐weighing technique method during 45‐min feeding bouts; evaluate the degree of agreement between the two techniques; and to test the effect of different potential sources of variation on the reliability of the CBM. The CBM method has been used to describe the intake behavior of grazing herbivores. In this study, we evaluated a new approach to this method, that is, whether it is a good proxy for determining the intake of grazing animals. Three experiments with grazing sheep were carried out in which we tested for different sources of variations, such as the number of observers, level of detail of bite coding grid, forage species, forage allowance, sward surface height heterogeneity, experiment site, and animal weight, to determine the short‐term intake rate (45 min). Observer (*P_exp1_* = 0.018, *P_exp2_* = 0.078, and *P_exp3_* = 0.006), sward surface height (*P_exp2_* < 0.001), total number of bites observed per grazing session (*P_exp2_* < 0.001 and *P_exp3_* < 0.001), and sward depletion (*P_exp3_* < 0.001) were found to affect the absolute error of intake estimation. The results showed a high correlation and agreement between the two methods in the three experiments, although intake was overestimation by CBM on experiments 2 and 3 (181.38 and 214.24 units, respectively). This outcome indicates the potential of CBM to determining forage intake with the benefit of a greater level of detail on foraging patterns and components of the diet. Furthermore, direct observation is not invasive nor disrupts natural animal behavior.

## Graphical abstract

We showed an agreement between the Continuous bite Monitoring method and Double‐Weighing Technique method; Continuous Bite Monitoring method is a good proxy for determining the intake of grazing animals; Direct observation is not invasive nor disrupts natural animal behavior.

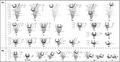

## 1 INTRODUCTION

One of the most important processes influencing the ecology of mammalian herbivores is how vegetation structure and composition affect dry mater and nutrient intake rate. Since herbivores complete thousands of bites per day, processes regulating the formation of a bite and resulting intake rate have tremendous repercussions on animal and plant ecology (Shipley, 2007). In domestic herbivore production, forage intake is well known to be the most important component affecting performance or productivity (Illius & Gordon, 1987). For wild mammalian herbivores, measurement of short‐term intake is essential on the study of energy balance between forage intake, exploration and displacement (e.g., Charnov, 1976), functional responses (e.g., Durant et al., 2003; Smallegange & Brunsting, 2002), habitat selection (Courant & Fortin, 2012), and coexistence between herbivores species (Tilman & Borer, 2015).

Yet, the estimation of short‐term intake rate (STIR) in grazing herbivores remains one of the biggest methodological challenges in animal science studies (Garnick et al., 2018; Mayes & Dove, 2000). At a daily scale, the use of plant markers, particularly n‐alkanes (Dove & Mayes, 2006) is an accurate method to estimate individual forage intake, apparent digestibility, and portion of diet composition under grazing conditions (Barcia et al., 2007; Gordon, 1995; Mayes & Dove, 2000). However, it requires intensive animal manipulation and labor for dosing, fecal sample collection, processing, and laboratory extraction (González‐García et al., 2017). Multiple other methods involving acoustic (e.g., Galli et al., 2011, 2017) or movement/inertia (e.g., Andriamasinoro et al. 2017; Rayas‐Amor et al., 2017) sensors have been tested to various levels of success on discriminating jaw movements (i.e., bite, chew, rumination). Walk‐over weighing devices (González‐García et al., 2017) and the “RumiWatch System” (Rombach et al., 2018; Ruuska et al., 2016) were developed for long‐term forage intake studies. For determining STIR, the double‐weighing technique (Penning & Hooper, 1985) is one of the most used procedures (Giovanett et al., 2017). It estimates the amount of forage intake in grazing sessions by the difference of animal weight pre‐ and postgrazing. Another previously employed methodology, esophageal fistulation (Geremia et al., 2018; Stobbs, 1973a, 1973b), is extremely invasive and not practical in rangeland situations.

Although those methods provide valuable information, the grazing process is described in terms of number and distribution of jaw movements, total intake, or average bite mass, bite rate, and intake rate over entire grazing sequences or days. They offer little detail of the dynamics of foraging actions and do not infer about variations of these variables as a function of local differences in the vegetation structure and composition (Bonnet et al., 2015). Herbivores respond to the botanical and structural diversity of grasslands, making decisions on choices and combinations of forages harvested at different space‐time scales (Provenza et al., 2015). Evaluating in detail the components of a diet is an essential factor for many studies of plant–animal interaction.

Hand plucking (Cook, 1964; Halls, 1954) has been used as a simple and cheap alternative for simulating intake and diet selection by wild (Collins & Urness, 1983; Hudson & Frank, 1987; Okello et al., 2002; Renecker & Hudson, 1985) and domestic herbivores (Agreil & Meuret, 2004; Agreil et al., 2006; Hobbs et al., 1983). By using similar principles, the continuous bite monitoring (CBM) method proposed by Agreil and Meuret (2004) represents a useful tool for a complete description of the foraging activities and grazing environment at the bite level. The advantages of the CBM method over other methods rely on real‐time recording of detailed descriptions of all foraging activities performed by the animal, bites taken in each plant species or structure, the estimation of the nutritional value of the plant tissue corresponding to each bite code from each plant species, and the exploration of the dynamics of the animal feeding station behavior (Azambuja Filho et al., 2020; Bolzan et al., 2020; Bonnet et al., 2015; Molnár et al., 2020; Torres‐Fajardo et al., 2019). Thus, it allows for a great level of detail in the description of the foraging process. On the other hand, it is known that the CBM method requires the progressive training of the observer and has a certain level of bias depending on experience and dedication (Bonnet et al., 2011).

Previous studies also questioned the reliability of the method as a function of different variables such as vegetation structure, period and duration of the observation and the level of detail of bite coding grid (Bolzan et al., 2020; Bonnet et al., 2015). Based on a pre‐evaluation of available vegetation and observation of diet selection and grazing process, possible “bites” are classified into categories of bite codes (BC) to compose a reference grid of foraging activities (i.e., BC, grazing, ruminating, and resting). Observers then monitor target animals for a determined period to record where all activities are registered. Observed BC taken are then simulated (i.e., hand‐plucked) to estimate the mass and nutritive value of each bite for further calculation of intake. Therefore, our objective was as follows: (a) to evaluate the reliability of the CBM method in determining herbage intake in grazing sheep compared to the standard double‐weighing technique (DW) method during 45‐min feeding bouts, (b) evaluate the degree of agreement between the two techniques, and (c) to test the effect of different potential sources of variation (observer and animal identity, vegetation structure and period and duration of the observation) on the reliability of the CBM.

## 2 MATERIALS AND METHODS

### 2.1 Site, treatments, and experimental design

**FIGURE 1:**
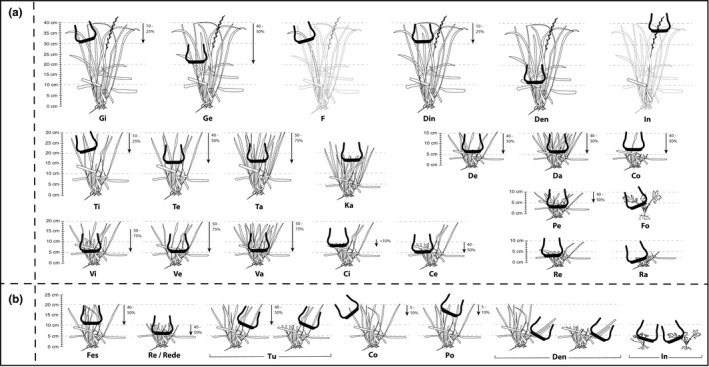
Representation of the types of sheep bites with their respective codes used in the Continuous Bite Monitoring method in experiment 1 (a) with Italian ryegrass, 2 and 3 (b) with Tall fescue. Drawing, in experiment 2 and 3 (b), represents the codes used in all tested heights, here demonstrated for the height of 20 cm. *Note*: The same codes were used for the other treatments. The arrows represent the depth of the bite in bite type. Description of bites is in Table 1 in Supporting Information

Three independent studies were conducted in which we tested for different sources of variation: number of observers, level of detail of BC grid (Figure 1), forage species, forage allowance, sward surface height (SSH) heterogeneity, experiment site, and animal breed. Experiment 1 was carried out between 15 September and 15 October 2014, on an area of approximately 0.50 ha of self‐seeding Italian ryegrass (*Lolium multiflorum* Lam.) at the experimental farm of the Federal University of Rio Grande do Sul, Brazil (30°05′27″S, 51°40′18″W). The area was divided into two paddocks of 0.25 ha (experimental areas) with salt and water freely available. In this protocol, there were no defined criteria for managing the sward pregrazing structure. Only average SSH measurements were collected at the time of observation.

Experiments 2 and 3 were carried out at the Canguiri experimental station of the Federal University of Paraná, Brazil (25°26′30″S and 49°7′30″W). The experiments were established in a 0.3 ha experimental area of tall fescue cv. INIA Aurora (*Schedonorus arundinaceus* [Schreb.] Dumort) sown in June 2015 on a prepared seedbed, at 55 kg/ha. Beginning in September 2015, the experimental area was managed under continuous stocking with SSH maintained between 10 and 15 cm, except just prior to and during the grazing events when the different pre‐grazing SSH treatments were imposed. Experiment 2 was carried out between 24 June and 12 July 2016, and experiment 3 was carried out between 15 and 24 November 2016. In experiment 2, five homogeneous pre‐grazing SSH (14, 17, 20, 23, and 26 cm) were evaluated in a randomized complete block design with four replicates. In experiment 3, five levels of depletion (0, 20, 40, 60, and 70%) of average SSH (20 cm) were evaluated in a randomized complete block design with four replicates, through grazing with nonexperimental animals prior to grazing sessions to measure behavior. A more detailed description management protocol can be found in Szymczak et al. (2020).

### 2.2 Sward measurements

Five hundred SSH measurements were taken in the two experimental paddocks of experiment 1 to characterize the vegetation structure. Mean SSH was 38.0  (±13.12) and 38.7  (±12.98) cm for paddocks 1 and 2, respectively. For experiments 2 and 3, 150 points pre‐grazing SSH within each sampling unit were measured. Pre‐grazing SSH were 14.2 (±0.19), 17.3 (±0.20), 19.7 (±0.27), 22.8 (±0.28), and 25.9 (±0.26) cm, for treatment of 14, 17, 20, 23, and 26 cm in experiment 2, respectively. The measured pre‐grazing SSH were 20.2 (±0.18), 16.5 (±0.52), 12.2 (±0.52), 8.3 (±0.48), and 5.9 (±0.37) cm, for treatments 0, 20, 40, 60, and 70% of depletion in experiment 3, respectively.

### 2.3 Intake and grazing behavior evaluations

#### 2.3.1 Animals and experimental procedures

Procedures involving the experimental animals were conducted under the Guidelines for the Use of Animals (2012) and complied with ethical guidelines published by the International Society for Applied Ethology. All procedures involving animals were approved by the Commission for Ethics in the Use of Animals of the Sector of Agricultural Sciences of the Federal University of Paraná (024/2016).

Two methodologies were used simultaneously during grazing tests to measure short‐term intake rate (STIR): the double‐weighing technique (DW) as the reference practice (Penning & Hooper, 1985) and the continuous bite monitoring (CBM) method (Agreil & Meuret, 2004; Bonnet et al., 2015). In experiment 1, eight Texel ewes (42.07 ± 3.15 kg LW) were used. Sixty days before the data collection, ewes were allocated on an adjacent Italian ryegrass pasture for acclimation to forage and adaptation to observers and equipment. During the experimental procedure, animals were distributed in two groups of four testers per paddock, where two testers per paddock were used for evaluation of the CBM and DW. After the evaluations, animals were placed back on the adjacent pasture for the remaining of the day. In experiments 2 and 3, six White Dorper x Suffolk ewes were used with an average weight of 61.9 ± 5.5 kg. Two animals were chosen as testers, all previously adapted to the experimental procedure and maintained in an area similar and adjacent to the experimental paddocks.

#### 2.3.2 Continuous bite monitoring

Experiment 1 involved three observers: one with previous experience on the methodology with wild herbivores and cattle (EO; Bonnet et al., 2015), and two new observers (TO1 and TO2) were trained by EO. Experiments 2 and 3 involved four different observers, all inexperienced. Prior to the beginning of the experiments, a mutual familiarization phase was adopted for three weeks. During this phase, animals were handled daily to acclimate to observers and protocols and for observers to familiarize with pasture and grazing behavior. Once the tester animals were identified and familiarized with the evaluators, all bites observed were described and classified into categories based on the observation of the animals' intake behavior before the experiments under the following aspects: (a) structural and nutritional distribution of the components in the sward; (b) the nature, size, density, and position of selected plant parts by animals, as a set of leaves, isolated leaves or inflorescences; and (c) handling (gathering herbage into the mouth, severing the herbage, ingestive mastication, and swallowing, Laca et al., 1994). Simple codes were established for each bite category agreed upon by all observers, composing a grid of codes for the identification of bites in real time (Figure 1a,b). The level of detail for each BC grid differed based on SSH, phenological stages, plant density heterogeneity, and species diversity. For experiments 2 and 3, the same grid was used, but the dimensions and masses varied (Annex 1 and 2).

After three weeks of training, both experienced and naïve observers were able to codify with confidence every bite observed (Bonnet et al., 2011). The three observers in experiment 1 (one observer per tester animal per paddock in each session evaluated) and four observers on experiments 2 and 3 (two observers per experiment, one observer per tester animal, and one tester per paddock) collected data by standing close to the animals (within 1 m), during 45‐min grazing sessions. Thirty‐two (experiment 1) and twenty (experiments 2 and 3) sessions were conducted. These sessions were blocked into morning and afternoon periods, arranged in a completely randomized design. After each grazing session had finished and while the animals remained in a common pen for determining insensible weight losses, each bite category was simulated (minimum 22 hand‐plucks for each bite type, for BC more frequent we replicated the samples). Samples were collected in paper bags and placed in a thermal box and weighed immediately after collection to estimate fresh matter (FM) intake. Intake was calculated as a sum of FM for all recorded bites. Data were registered using a Sony ICD‐PX312 (Sony Corp., Japan) digital voice recorder and, subsequently, transcribed using the J Watcher software (www.jwatcher.ucla.edu).

#### 2.3.3 Short‐term intake rate

During the experimental period each day around 5:40 a.m. (experiment 1) or 6:30 a.m. (experiments 2 and 3), animals were moved to the handling area, fitted with harnesses for a total collection of urine and feces, and weighed at *t_1_* (*W_1_* = initial weight for estimating the rate of insensitive weight losses (H_2_O evaporation, CO_2_ and CH_4_ losses); RIWL pre‐grazing). After being weighed, the animals remained in a common pen for 45 min without access to feed or water and then weighed again at *t_2_* (*W_2_* = final weight for pregrazing RIWL and pre‐grazing weight). Immediately after, all the animals were conducted and allotted to their paddocks for the 45‐min grazing session (*ET*). Once the grazing session was finished, the animals were led to the handling area and the tester animals were weighed at *t_3_* (*W_3_* = post‐grazing weight and initial weight for the post‐grazing RIWL). The tester animals then remained in a common area without access to feed, water, or shade for 45 min until being weighed at *t_4_* (*W_4_* = final weight for post‐grazing RIWL). The harnesses were then immediately removed, and the animals returned to the adjacent area. This same procedure was repeated in the afternoon (between 2:15 p.m. and 6:30 p.m. for experiment 1 and between 2:30 and 6:30 p.m. for experiments 2 and 3). The animals were weighed using an electronic scale (MGR‐3000 Junior, Toledo, Canoas, Brazil) with a capacity of 200 kg (5‐g increments). Short‐term intake rate (g FM min^−1^; Equation 1) was calculated by measuring the weight change, corrected for insensible weight loss, and the time spent grazing, according to Penning and Hooper (1985). Total FM intake assessed by DW was calculated by multiplying STIR and ET.

(1) $$\text{STIR}=\left\{\left[\frac{\left(W_{2}‐W_{1}\right)}{\left(t_{2}‐t_{1}\right)}\right]+\left[\frac{\left(W_{3}‐W_{4}\right)}{\left(t_{4}‐t_{3}\right)}\right]\right\}\times \left[\frac{\left(t_{2}‐t_{1}\right)}{\text{ET}}\right]$$

### 2.4 Statistical analysis

The data were analyzed using the R software (R Development Core Team, 2016). Animal test group was the experimental unit. We systematically verified normality and homogeneity of the residuals. Pearson correlation was used as mean accuracy between the methods and was considered poor (<0.4), reasonable (0.4 to 0.6), good (0.6 to 0.8), or excellent (0.8 to 1.0). Bland–Altman plots were created to indicate the degree of agreement between the two techniques (Bland & Altman, 1999). The limits of agreement were determined by calculating the bias and standard deviation of the paired differences. The standard deviation was multiplied by the 1.96 quantiles of a normal distribution, and then, the amount of the calculated average was added or subtracted to provide the upper or lower limits, respectively. Thus, the agreement limits were calculated as bias ± standard deviation. One‐sample t‐ test, at a significance level of 95%, was performed to check if there was a significant difference from zero, for the comparison between the methods.

## 3 RESULTS

**FIGURE 2:**
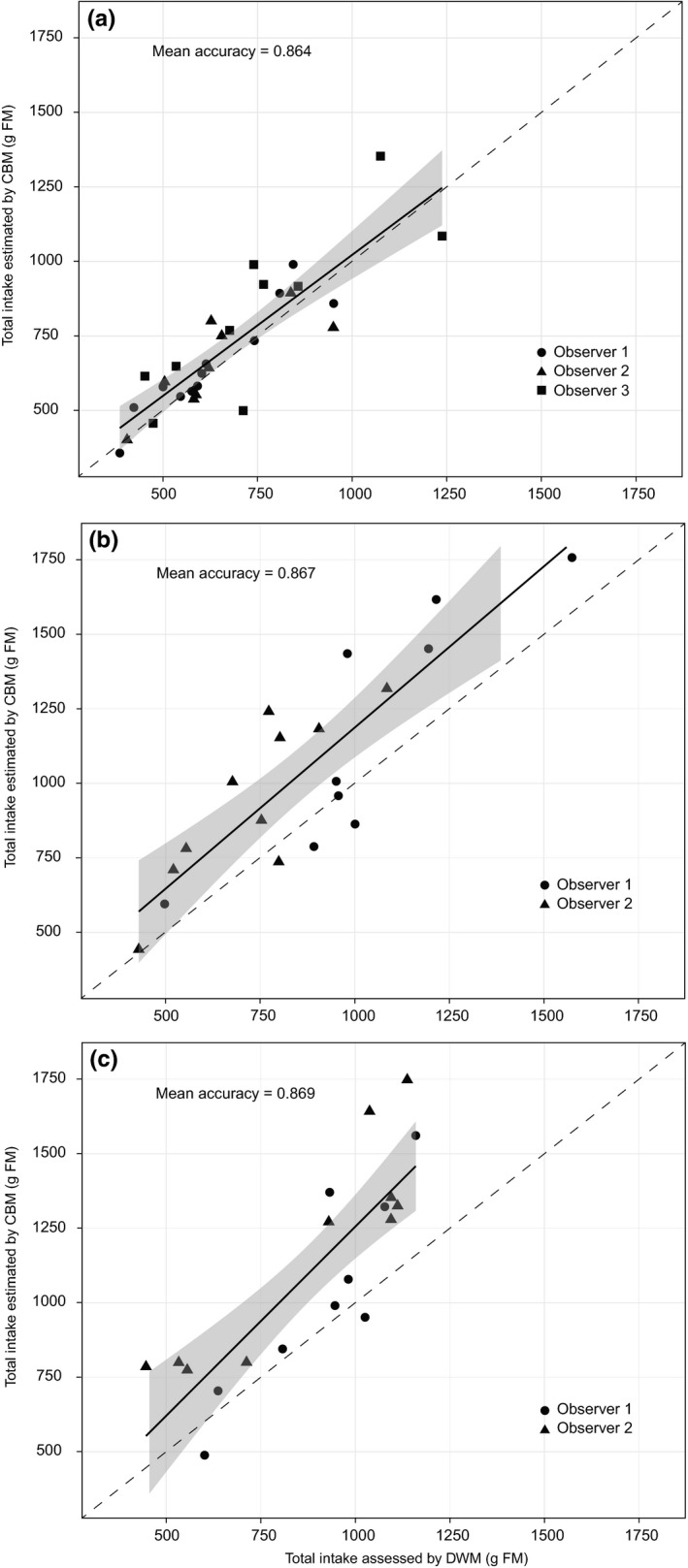
Relationship between the total intake of fresh matter (g FM) of ewes during grazing sessions of 45 min estimated through continuous bite monitoring assessed by the double weight technique, in experiment 1 (a), 2 (a) and 3 (C). Solid line represents the linear model between the two methods (*p* <.001), dashed lines represents identity (*Y* = *X*), and gray area represent the confidence interval of the measurement through double weight with regard to scale precision

The correlation between estimated forage intake (as FM) through the CBM and DW methods for ewes in 45‐min grazing sessions is presented in Figure 2. The overall mean correlation over 32 observations was 0.864, in experiment 1 (Figure 2a), and over 20 observations were 0.867 and 0.869, in experiment 2 and 3, respectively (Figure 2b,c). A significant effect of the observer on the absolute error of intake estimation (Table 1) was found in experiment 1. In experiments 2 and 3, we found significant effects of sward structure (SSH and sward depletion, respectively), observer and the total number of bites observed per grazing session (Table 1). Day of measurement, individual animals or period of the day had no significant effect on the absolute error in none of the experiments (Table 1).

**TABLE 1:** ANOVA table for the potential sources of variation of the error in the estimation of fresh matter intake through CBM. *Day* refers to the number of days with observation since the beginning of the experiment, *Period* to the period of the day evaluated (morning or afternoon) and *Total bites* to the total number of bites observed during one trial. Interactions were not significant and were removed from the final model

Source of variation	*df*	*F* value	*p* value
Experiment 1			
Observer	2	4.75	.018
Animal	3	1.97	.14
Day	1	0.06	.81
Total bites	1	0.17	.68
Experiment 2			
Sward surface height	4	12.478	.0002
Observer	1	3.633	.078
Day	4	1.091	.314
Period	1	0.083	.777
Total bites	1	27.927	.0000
Experiment 3			
Sward depletion	4	91.46	.0000
Observer	1	11.02	.006
Day	4	7.724	.101
Period	1	1.640	.68
Total bites	1	15.392	.0000

**FIGURE 3:**
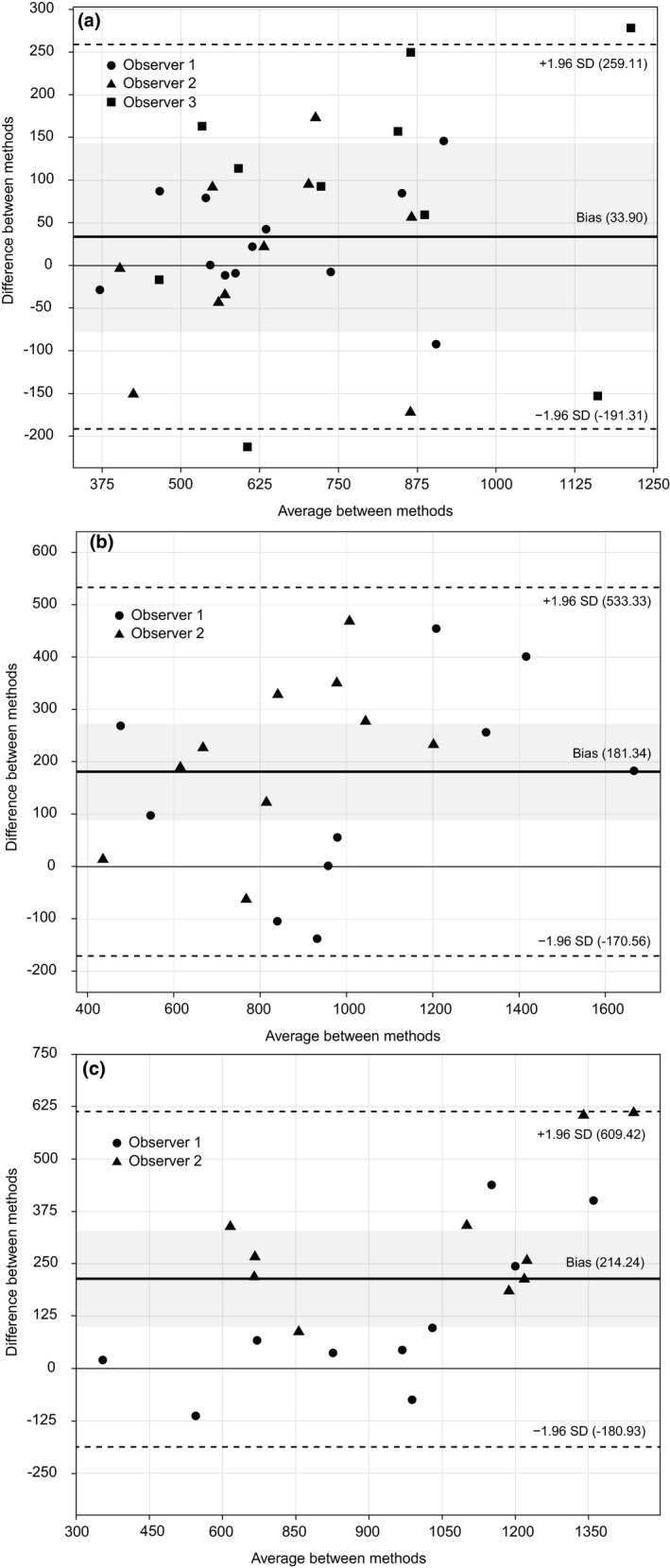
Bland–Altman plots showing the paired differences against the average between CBM and DWM methods in experiments 1 (a, *p* =.1052 in one‐sample *t* test), 2 (b, *p* =.0002 in one‐sample *t* test), and 3 (c, *p* =.0001 in one‐sample *t* test). Mean bias is represented by black line and limits of agreement are shown by the dashed lines, while confidence intervals are shown by the gray areas

The Bland–Altman analysis (Figure 3) showed the bias between methods of 33.90, 181.38, and 214.24 g FM, and the limits of agreement: 259.11 and −191.31, 533.33, and −170.56 and 201.62, and −180.93 g FM, for experiment 1, 2, and 3, respectively. The bias value obtained in the comparison of the methods means that on average the CBM method measures 33.90, 181.38, and 214.24 more units in relation to the DW method, for experiments 1, 2, and 3, respectively. There was a significant difference between zero and bias by one‐sample *t* test, only for experiments 2 (*p* =.0002) and 3 (*p* =.0001).

## 4 DISCUSSION

Direct observation has a large capacity for detailed assessment of the grazing processes, considering important factors at the plant–animal interface. Bonnet et al. (2011), using cows (*Bos taurus taurus* L.) and goats (*Capra hircus* L.), showed that the ability of different observers to evaluate short‐term intake after training had a correlation greater than 85%. Similarly, our results showed correlation topping 86.4% in comparison with the standard DW technique (Penning & Hooper, 1985). However, we found significant differences in the estimate of intake between the two methodologies in experiments 2 and 3, with generally overestimation of intake by the CBM regarding to the DW (Figure 2b,c). Given the differences in sward pasture conditions and assuming all observers received similar levels of training, this could indicate that a greater detail of bite types in the description of the grazing process could improve the accuracy of the method.

Differences in estimation of intake between the two techniques may be associated with inherent error of both methodologies. Many studies in the literature reported high interindividual variability when using the DW method (Fonseca et al., 2012; Guzatti et al., 2017; Mezzalira et al., 2017). Other sources of variation, such as differences between paddocks and shifts (a.m. vs. p.m.) also add to the compound variation for many of the methodologies used for estimating intake (Bailey et al., 1996; Fraser, 2009; Gregorini, 2012). For example, Lukuyu et al. (2014) compared two pasture disappearance‐based techniques (rising‐plate meter and capacitance meter) and two chemical marker‐based techniques (dosed n‐alkanes and chromic oxide) techniques of forage intake in steers, showing high internal variation (coefficients of variation of 28% for the capacitance meter and 44% for the plate meter) in estimates and low correlation (*r* = .51) between chromic oxide and the plate meter. They found no correlation between disappearance‐based and alkane methods. Greenwood et al. (2014) found correlations between the biomass disappearance and C32/C31 and C32/C33 n‐alkane of 0.77 and 0.70, respectively.

The correlation analysis (Figure 2) shows only of the strength of relationship between the variables but not the agreement between them (Giavarina, 2015). A high correlation between the methods can mostly come from the large range in fresh matter intake observed during the experiment (Giavarina, 2015). The Bland–Altman analysis shows the agreement between the methods and is a parameter of greater consistency to compare techniques (Giavarina, 2015; Myles, 2007), as it is evaluated according to the data dispersion. In cases of good agreement, the scattering of points is diminished and points lie relatively close to the solid, bold line (mean bias; Figure 3) (Giavarina, 2015; Myles, 2007). Our results had a high dispersion; however, points were mostly within the limits of agreement in all cases. This denotes agreement between methods, but with high variability in measuring intake. In addition, the Bland–Altman analysis demonstrated an intake overestimation measures for CBM compared to DW method, mostly in experiments 2 and 3 (Figure 3b,c). It is important to point out that the observer's interpretation of the animal's action in the execution of the bite, along with the factors that establish the bite category (i.e., the type of tissue, position in the sward and density), are determinants for the accuracy of the simulation. Therefore, the smaller number of bite codes on experiments 2 and 3 resulted in a greater range of bite mass for each of the BC, increasing the dispersion of the data which resulted in the overestimation of intake with CBM. Alternatively, a more detailed assessment (i.e., experiment 1) dilutes the variations of the effects by the pasture structure (SSH and sward depletion), observer and the total number of bites in multiple BC (Bolzan et al., 2020), minimizing the difference between methods (Table 1).

Evaluating foraging behavior at the smallest scale of the grazing process, the bite (Laca & Ortega, 1995), allows us to understand each bite type during the grazing process (Illius & Gordon, 1987). It elucidates the spatio‐temporal distribution and variability of the grazing process in response to the variation in components of the vegetation structure. Our work provides evidence of the potential and limitations of the CBM technique. This tool can be used with great assurance in the estimations of STIR, considering the influence factors such as pasture structure (Allden & Whitakker, 1970; Laca et al., 1992; Mezzalira et al., 2017; Nadin et al., 2019), digestibility (Drescher et al., 2006), and selectivity (Hodgson, 1979). Both the level of observer knowledge of pasture science principles (i.e., understanding of pasture structure and botanical composition in diverse grasslands) and level of training observers receive are limiting factors for the success of this direct observation technique. The posed question to be addressed in the study directly conditions the detailing of the description of the food actions (BCs) to be evaluated, as well as other ethological standards. In addition to the intake rates, we were able to know the fraction of each BC regarding what they eat, how they eat, and how much they eat of each item.

### 4.1 Implications

Our results indicate the accuracy of the hand plucking method and CBM as an alternative to quantify forage intake, that is, there was agreement between the studied methods. We found an overestimated consumption when using CBM in comparison with DW in experiments 2 and 3. However, we hypothesize that the difference between the methods can decrease by increasing the detail of the BC grid. This extends the possibilities of evaluating animals during the foraging process, especially free‐ranging, without significant modifications on the environment or animal manipulation. With the knowledge of quantitative reliance, we have the potential to complement other methodologies, sensor calibration, and subsequent use in long‐term evaluations. This reality would increase the evaluation capacity of several animals at the same time, in comparison with the CBM method, which restricts the evaluation of only one animal per observer over time, which represents a large time cost in training, evaluation, and transcription.

There is a high demand from the scientific community and general society for experimental protocols that promote animal welfare (Driscoll & Bateson, 1988). Noninvasive methodologies are extremely important to preserve natural animal behavioral principles, avoid diseases (e.g., chromium oxide possesses carcinogenic properties; Sedman et al., 2006) or injuries, and not alter the affective states of animal (avoid pain, fear, suffering, frustration, and distress) (Driscoll & Bateson, 1988; Fraser, 2009; Sherwin et al., 2003). We believe in the potential of the CBM methodology as an important alternative because it does not require physical contact, adaptation to unnatural conditions, or the use of equipment coupled to the animal. Thus, it has high potential for reproducing grazing animal intake in different environments and situations while maintaining animal welfare.

## CONFLICT OF INTEREST

None declared.

## AUTHOR CONTRIBUTIONS

**Anderson Michel Soares Bolzan**: Conceptualization (Equal); Data curation (Equal); Formal analysis (Equal); Methodology (Equal); Writing‐original draft (Equal). **Leonardo Silvestri Szymczak**: Data curation (Equal), Formal analysis (Equal), Methodology (Equal), Writing‐original draft (Equal). **Laura Nadin**: Data curation (Equal), Formal analysis (Supporting), Investigation (Equal), Methodology (Supporting), Writing‐original draft (Equal). **Olivier François Bonnet**: Conceptualization (Equal), Data curation (Equal), Formal analysis (Equal), Investigation (Equal), Methodology (Equal), Writing‐original draft (Equal). **Marcelo Osorio Wallau**: Formal analysis (Equal), Writing‐original draft (Equal). **Anibal de Moraes**: Investigation (Supporting), Methodology (Supporting), Supervision (Lead), Writing‐original draft (Supporting). **Renata Franciéli Moraes**: Data curation (Supporting), Formal analysis (Supporting), Methodology (Supporting), Writing‐original draft (Supporting). **Alda Lucia Gomes Monteiro**: Data curation (Supporting), Project administration (Lead), Supervision (Supporting), Writing‐original draft‐(Supporting). **Paulo César de Faccio Carvalho**: Conceptualization (Equal), Data curation (Supporting), Methodology (Supporting), Project administration (Lead), Supervision (Lead), Writing‐original draft (Lead).

## Supporting information

Supplementary Material

## Data Availability

These data are available at https://doi.org/10.5061/dryad.573n5tb73
