# Supplementary material for: What, how, and how much do herbivores eat? The Continuous Bite Monitoring method for assessing forage intake of grazing animals
Source: Ecol Evol. 2021 Jun 25;11(14):9217–26. doi: 10.1002/ece3.7477 (PMC8293712; doi:10.1002/ece3.7477)
Supplement: Supplementary file 1 — Supplementary Material [file ECE3-11-9217-s001.pdf]

What, how and how much do herbivores eat? The Continuous Bite Monitoring method for assessing forage intake of grazing animals.

Supporting Information

Annex1. Description of the types of sheep bites with their respective codes used in the continuous monitoring of bite method in Experiment 1, 2 and 3, represented in the Figure 1.

Bite Category Code	Bite Category Description
	Experiment 1
Gi	Superficial bite 10 - 25 % in sward 40 cm
Ge	Bite with depth 40 - 50 % in sward 40 cm
Din	Superficial bite 10 - 25 % of sparse leaves in sward 40 cm
Den	Bite of exploration in sward 40 cm
Ti	Superficial bite 10 - 25 % of sparse leaves in sward 30 cm
Te	Bite with depth 40 - 50 % in sward 30 cm
Ta	Dense bite of leaves with depth 50 - 75 % in sward 30 cm
Vi	Bite with depth 50 - 75% in sward 15 cm
Ve	Bite of sparse leaves with depth 50 - 75 % in sward 20 cm
Va	Dense bite of leaves with depth 50 - 75 % in sward 20 cm
De	Bite with depth 40 - 50% in sward 15 cm
Da	Dense bite of leaves with depth 40 - 50% in sward 15 cm
Ci	Superficial bite < 10 % in sward 10 cm
Ce	Bite with depth 40 - 50% in sward 10 cm

Co	Bite of plant stalks
Ra	Bite in sward grazed < 8 cm
Re	Bite in sward < 8cm
Fo	Bite in other plants or plants parts (i. e. *Sida rhombifoli*a L.)
In	Bite in inflorescences of Italian ryegrass
Ka	Bite superficial mixte in leaves grazed and ungrazed in sward 20 cm
F	Single leaf Italian ryegrass
Experiment 2	
Fes[1]*	Bite with greater mass compared to the others. Bites depth corresponding to 40 to 50% of the sward surface height of the tall fescue (first grazing horizon) at the sward surface height of 14 cm.
Fes[2]	Bite with greater mass compared to the others. Bites depth corresponding to 40 to 50% of the sward surface height of the tall fescue (first grazing horizon) at the sward surface height of 17 cm.
Fes[3]	Bite with greater mass compared to the others. Bites depth corresponding to 40 to 50% of the sward surface height of the tall fescue (first grazing horizon) at the sward surface height of 20 cm.
Fes[4]	Bite with greater mass compared to the others. Bites depth corresponding to 40 to 50% of the sward surface height of the tall fescue (first grazing horizon) at the sward surface height of 23 cm.
Fes[5]	Bite with greater mass compared to the others. Bites depth corresponding to 40 to 50% of the sward surface height of the tall fescue (first grazing horizon) at the sward surface height of 26 cm.
Tu[1]	Bite similar to FES but with less mass. Bites depth corresponding to 40 to 50% of the sward surface height of the tall fescue (first grazing horizon) at the sward surface height of 14 cm.
Tu[2]	Bite similar to FES but with less mass. Bites depth corresponding to 40 to 50% of the sward surface height of the tall fescue (first grazing horizon) at the sward surface height of 17 cm.
Tu[3]	Bite similar to FES but with less mass. Bites depth corresponding to 40 to 50% of the sward surface height of the tall fescue (first grazing horizon) at the sward surface height of 20 cm.

Tu^{4}	Bite similar to FES but with less mass. Bites depth corresponding to 40 to 50% of the sward surface height of the tall fescue (first grazing horizon) at the sward surface height of 23 cm.
Tu^{5}	Bite similar to FES but with less mass. Bites depth corresponding to 40 to 50% of the sward surface height of the tall fescue (first grazing horizon) at the sward surface height of 26 cm.
Den^{1}	Bite of exploration of tall fescue leaves from lower strata or close to the ground at the sward surface height of 14 cm.
Den^{2}	Bite of exploration of tall fescue leaves from lower strata or close to the ground at the sward surface height of 17 cm.
Den^{3}	Bite of exploration of tall fescue leaves from lower strata or close to the ground at the sward surface height of 20 cm.
Den^{4}	Bite of exploration of tall fescue leaves from lower strata or close to the ground at the sward surface height of 23 cm.
Den^{5}	Bite of exploration of tall fescue leaves from lower strata or close to the ground at the sward surface height of 26 cm.
Po^{1}	Exploration bite of 4 to 2 leaf tips at the sward surface height of 14 cm. Bite with low mass.
Po^{2}	Exploration bite of 4 to 2 leaf tips at the sward surface height of 17 cm. Bite with low mass.
Po^{3}	Exploration bite of 4 to 2 leaf tips at the sward surface height of 20 cm. Bite with low mass.
Po^{4}	Exploration bite of 4 to 2 leaf tips at the sward surface height of 23 cm. Bite with low mass.
Po^{5}	Exploration bite of 4 to 2 leaf tips at the sward surface height of 26 cm. Bite with low mass.
Co^{1}	Exploration bite of 1 leaf tip at the sward surface height of 14 cm. Bites with less mass compared to the others.
Co^{2}	Exploration bite of 1 leaf tip at the sward surface height of 17 cm. Bites with less mass compared to the others.
Co^{3}	Exploration bite of 1 leaf tip at the sward surface height of 20 cm. Bites with less mass compared to the others.

Co[4] Exploration bite of 1 leaf tip at the sward surface height of 23 cm. Bites with less mass compared to the others.

Co[5] Exploration bite of 1 leaf tip at the sward surface height of 26 cm. Bites with less mass compared to the others.

Re[1] Bite in places where they have already been grazed (second grazing horizon) within the same feeding station at the sward surface height of 14 cm. Bite made by the animal itself after intake the first grazing horizon.

Re[2] Bite in places where they have already been grazed (second grazing horizon) within the same feeding station at the sward surface height of 17 cm. Bite made by the animal itself after intake the first grazing horizon.

Re[3] Bite in places where they have already been grazed (second grazing horizon) within the same feeding station at the sward surface height of 20 cm. Bite made by the animal itself after intake the first grazing horizon.

Re[4] Bite in places where they have already been grazed (second grazing horizon) within the same feeding station at the sward surface height of 23 cm. Bite made by the animal itself after intake the first grazing horizon.

Re[5] Bite in places where they have already been grazed (second grazing horizon) within the same feeding station at the sward surface height of 26 cm. Bite made by the animal itself after intake the first grazing horizon.

Rede[1] Bite in places where they have already been grazed (second grazing horizon) outside of feeding station at the sward surface height of 14 cm. Bite performed in places grazed by another animal.

Rede[2] Bite in places where they have already been grazed (second grazing horizon) outside of feeding station at the sward surface height of 17 cm. Bite performed in places grazed by another animal.

Rede[3] Bite in places where they have already been grazed (second grazing horizon) outside of feeding station at the sward surface height of 20 cm. Bite performed in places grazed by another animal.

Rede[4] Bite in places where they have already been grazed (second grazing horizon) outside of feeding station at the sward surface height of 23 cm. Bite performed in places grazed by another animal.

Rede[5] Bite in places where they have already been grazed (second grazing horizon) outside of feeding station at the sward surface height of 26 cm. Bite performed in places grazed by another animal.

In[1] Bite in other species at the sward surface height of 14 cm.

In^2	Bite in other species at the sward surface height of 17 cm.
In^3	Bite in other species at the sward surface height of 20 cm.
In^4	Bite in other species at the sward surface height of 23 cm.
In^5	Bite in other species at the sward surface height of 26 cm.
	Experiment 3
Fes^A	Bite with greater mass compared to the others. Bites depth corresponding to 40 to 50% of the sward surface height of the tall fescue (first grazing horizon) at 0% depletion of pasture sward.
Fes^B	Bite with greater mass compared to the others. Bites depth corresponding to 40 to 50% of the sward surface height of the tall fescue (first grazing horizon) at 20% depletion of pasture sward.
Fes^C	Bite with greater mass compared to the others. Bites depth corresponding to 40 to 50% of the sward surface height of the tall fescue (first grazing horizon) at 40% depletion of pasture sward.
Fes^D	Bite with greater mass compared to the others. Bites depth corresponding to 40 to 50% of the sward surface height of the tall fescue (first grazing horizon) at 60% depletion of pasture sward.
Fes^E	Bite with greater mass compared to the others. Bites depth corresponding to 40 to 50% of the sward surface height of the tall fescue (first grazing horizon) at 70% depletion of pasture sward.
Tu^A	Bite similar to FES but with less mass. Bites depth corresponding to 40 to 50% of the sward surface height of the tall fescue (first grazing horizon) at 0% depletion of pasture sward.
Tu^B	Bite similar to FES but with less mass. Bites depth corresponding to 40 to 50% of the sward surface height of the tall fescue (first grazing horizon) at 20% depletion of pasture sward.
Tu^C	Bite similar to FES but with less mass. Bites depth corresponding to 40 to 50% of the sward surface height of the tall fescue (first grazing horizon) at 40% depletion of pasture sward.
Tu^D	Bite similar to FES but with less mass. Bites depth corresponding to 40 to 50% of the sward surface height of the tall fescue (first grazing horizon) at 60% depletion of pasture sward.

Tu^E	Bite similar to FES but with less mass. Bites depth corresponding to 40 to 50% of the sward surface height of the tall fescue (first grazing horizon) at 70% depletion of pasture sward.
Den^A	Bite of exploration of tall fescue leaves from lower strata or close to the ground at 0% depletion of pasture sward.
Den^B	Bite of exploration of tall fescue leaves from lower strata or close to the ground at 20% depletion of pasture sward.
Den^C	Bite of exploration of tall fescue leaves from lower strata or close to the at 40% depletion of pasture sward.
Den^D	Bite of exploration of tall fescue leaves from lower strata or close to the ground at 60% depletion of pasture sward.
Den^E	Bite of exploration of tall fescue leaves from lower strata or close to the ground at 70% depletion of pasture sward.
Po^A	Exploration bite of 4 to 2 leaf tips at 0% depletion of pasture sward. Bite with low mass.
Po^B	Exploration bite of 4 to 2 leaf tips at 20% depletion of pasture sward. Bite with low mass.
Po^C	Exploration bite of 4 to 2 leaf tips at 40% depletion of pasture sward. Bite with low mass.
Po^D	Exploration bite of 4 to 2 leaf tips at 60% depletion of pasture sward. Bite with low mass.
Po^E	Exploration bite of 4 to 2 leaf tips at 70% depletion of pasture. Bite with low mass sward.
Co^A	Exploration bite of 1 leaf tip at 0% depletion of pasture sward. Bites with less mass compared to the others.
Co^B	Exploration bite of 1 leaf tip at 20% depletion of pasture sward. Bites with less mass compared to the others.
Co^C	Exploration bite of 1 leaf tip at 40% depletion of pasture sward. Bites with less mass compared to the others.
Co^D	Exploration bite of 1 leaf tip at 60% depletion of pasture sward. Bites with less mass compared to the others.

Co^E	Exploration bite of 1 leaf tip at 70% depletion of pasture sward. Bites with less mass compared to the others.
Re^A	Bite in places where they have already been grazed (second grazing horizon) within the same feeding station at 0% depletion of pasture sward. Bite made by the animal itself after intake the first grazing horizon.
Re^B	Bite in places where they have already been grazed (second grazing horizon) within the same feeding station at 20% depletion of pasture sward. Bite made by the animal itself after intake the first grazing horizon.
Re^C	Bite in places where they have already been grazed (second grazing horizon) within the same feeding station at 40% depletion of pasture sward. Bite made by the animal itself after intake the first grazing horizon.
Re^D	Bite in places where they have already been grazed (second grazing horizon) within the same feeding station at 60% depletion of pasture sward. Bite made by the animal itself after intake the first grazing horizon.
Re^E	Bite in places where they have already been grazed (second grazing horizon) within the same feeding station at 70% depletion of pasture sward. Bite made by the animal itself after intake the first grazing horizon.
$Rede^A$	Bite in places where they have already been grazed (second grazing horizon) outside of feeding station at 0% depletion of pasture sward. Bite performed in places grazed by another animal.
$Rede^B$	Bite in places where they have already been grazed (second grazing horizon) outside of feeding station at 20% depletion of pasture sward. Bite performed in places grazed by another animal.
$Rede^C$	Bite in places where they have already been grazed (second grazing horizon) outside of feeding station at 40% depletion of pasture sward. Bite performed in places grazed by another animal.
$Rede^D$	Bite in places where they have already been grazed (second grazing horizon) outside of feeding station at 60% depletion of pasture sward. Bite performed in places grazed by another animal.
$Rede^E$	Bite in places where they have already been grazed (second grazing horizon) outside of feeding station at 70% depletion of pasture sward. Bite performed in places grazed by another animal.
In^A	Bite in other species at 0% depletion of sward surface height of the fescue.
In^B	Bite in other species at 20% depletion of sward surface height of the fescue.
In^C	Bite in other species at 40% depletion of sward surface height of the fescue.

In^D	Bite in other species at 60% depletion of sward surface height of the fescue.
In^E	Bite in other species at 70% depletion of sward surface height of the fescue.

* **X^Y model: X** represent the bite code category and ^{Y} represent the bite class, i.e. treatment within of experiment 2 and 3, different sward surface heights and sward depletion, respectively.

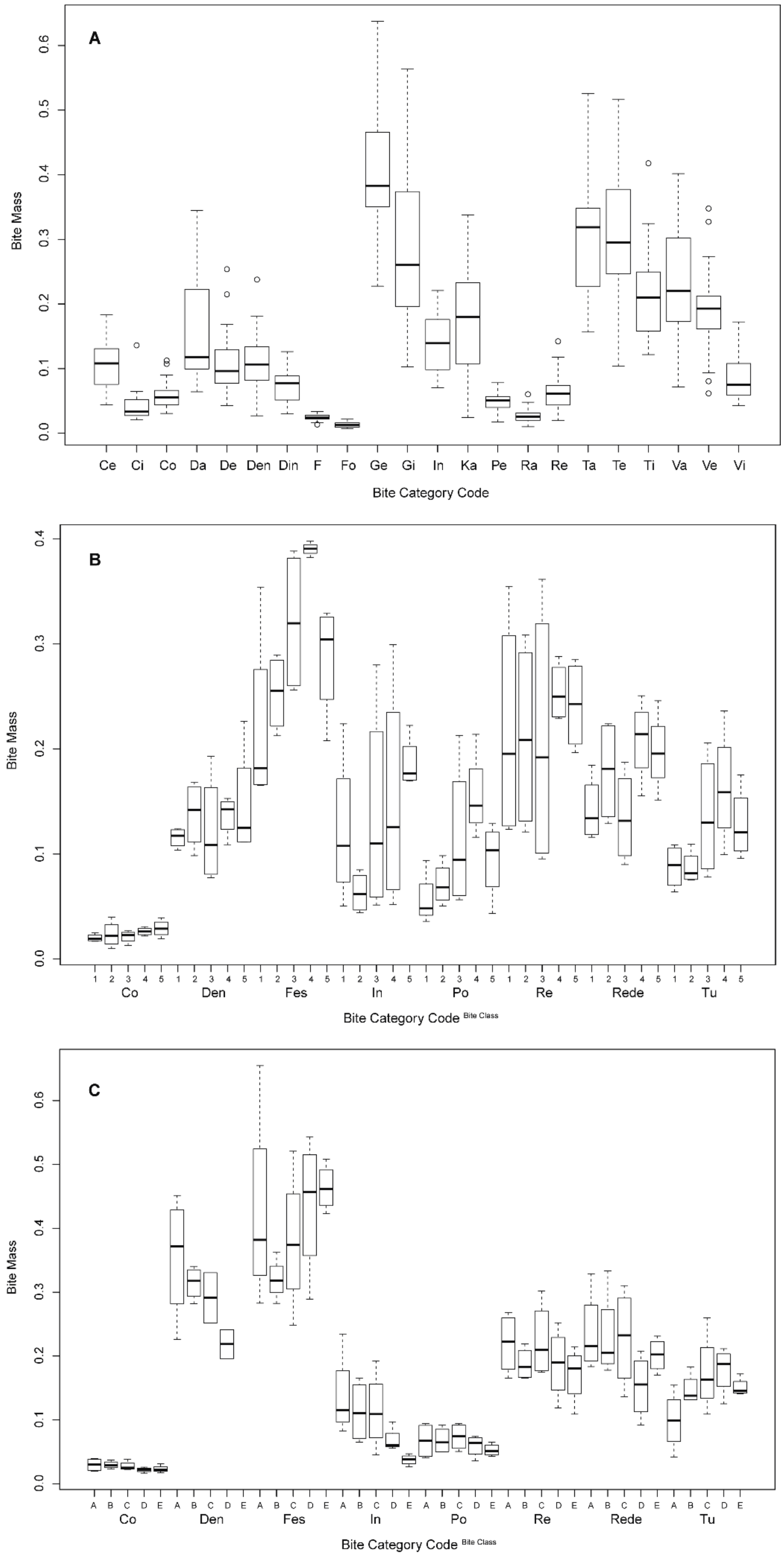

Annex 2. Distribution of bite mass (g DM) as estimated by hand-plucking by the 3 observers in experiment 1 (A), and 2 observers in experiment 2 (B) and 3 (C).

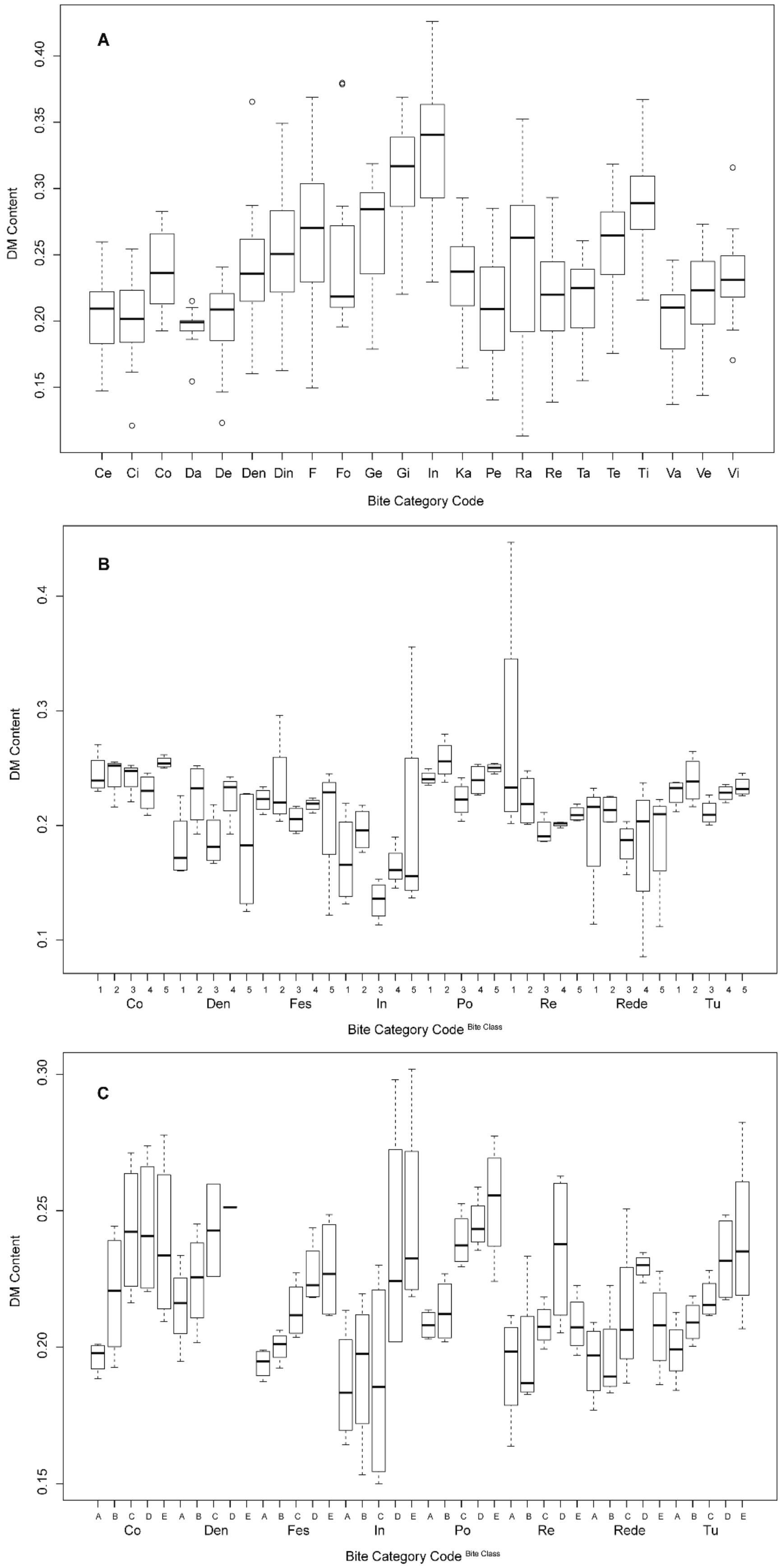

Annex 3. Dry matter (DM) content of bite-simulation samples collected during the early afternoon trial as a function of the bite category, in experiment 1 (A), 2 (B) and 3 (C).
